# Parental Autonomy Support, Parental Psychological Control and Chinese University Students’ Behavior Regulation: The Mediating Role of Basic Psychological Needs

**DOI:** 10.3389/fpsyg.2021.735570

**Published:** 2022-02-18

**Authors:** Songqin Wei, Timothy Teo, Anabela Malpique, Adi Lausen

**Affiliations:** ^1^Department of Psychology, University of Essex, Colchester, United Kingdom; ^2^Department of Educational Psychology, Faculty of Education, The Chinese University of Hong Kong, Hong Kong, China; ^3^School of Education, Edith Cowan University, Perth, WA, Australia; ^4^Department of Clinical Neuropsychology, Bamberg Hospital, Bamberg, Germany; ^5^Department of Mathematical Sciences, University of Essex, Colchester, United Kingdom

**Keywords:** parental autonomy support, parental psychological control, basic psychological need satisfaction, basic psychological need frustration, self-determination theory

## Abstract

The present research examined relationships between parental autonomy support, parental psychological control, and Chinese emerging adults’ autonomous regulation in their university studies as well as dysregulation in social media engagement. A total of 287 (102 female and 185 male) Chinese university students reported on their perceived parenting styles, psychological needs, and behavior regulation. Results showed that basic psychological need satisfaction was positively associated with parental autonomy support and autonomous regulation of learning; need frustration was positively correlated with parental psychological control and dysregulation in social media engagement. More importantly, psychological need frustration was a mediator of the relation between parental psychological control and dysregulation in social media engagement. Our findings suggest that students living in an autonomy-supportive familial environment tend to have satisfied psychological needs as well as autonomous learning behavior. Impairment of psychological needs could be one of the mechanisms through which psychologically controlling parenting was linked to dysregulation of social media use in Chinese culture.

## Introduction

Self-determination theory (SDT; Ryan and Deci, 2017), a broad framework for the study of human motivation, needs and well-being, posits that humans actively seek opportunities to satisfy their basic psychological needs for autonomy, competence, and relatedness. Specifically, *autonomy* refers to a volitional action that is manifested in the desire to maintain an integrated sense of self; *competence* is related to one’s capability to interact and behave in the environment; *relatedness* is described as the general sense of belonging and connecting to others. The satisfaction of these needs appears to be essential for optimal functioning and their alienation may undermine individual development (Deci and Ryan, 2000). However, low levels of psychological need satisfaction are different from the experience of need frustration (Vansteenkiste and Ryan, 2013; Chen et al., 2015; Costa et al., 2016). In other words, the former may reflect one lives in an environment that lacks needs support whereas the latter emphasizes one’s needs are directly and actively obstructed and could lead to severer consequences; therefore, these two dimensions should be tested distinctively. According to SDT, humans’ motivation to regulate their behavior is greatly driven by basic psychological needs and the satisfaction of these needs plays a vital role in positive adjustment and optimal functioning (Ryan and Deci, 2017). When needs are frustrated, the behavior is dysregulated and negative outcomes such as self-criticism, ill-being and even psychopathology would result (Bartholomew et al., 2011; Vansteenkiste and Ryan, 2013).

Ryan and Deci (2017) also emphasized that proximal social contexts such as families and peers may facilitate or obstruct the satisfaction of basic psychological needs and relate to different behavioral outcomes. Specifically, parental autonomy support (PAS) describes that parents actively encourage and support their children to be self-initiating and autonomous (Grolnick and Ryan, 1989). Autonomy-supportive parents help their children to explore self-value and interests with minimum control and pressure, thereby facilitating their psychological needs (Vansteenkiste and Ryan, 2013). Opposite to PAS, parental psychological control (PPC) refers to parenting behavior that is manipulative and intruding into children’s psychological world (Barber, 1996). Psychologically controlling parents usually use techniques such as guilt-induction, love withdrawal and shaming to regulate children’s behavior, which often thwarts their basic needs (Soenens and Vansteenkiste, 2010).

The present study attempted to analyze relations between parenting styles (i.e., autonomy support and psychological control), basic psychological needs and behavior regulation (i.e., learning and social media engagement) among Chinese university students. University students often experience significant transitions and challenges in life and their behavior and emotions are still affected by parents (Arnett, 2004). These challenges may be amplified within Chinese culture because of the longstanding cultural emphasis on academic success (Quach et al., 2015) and obedience to parents (Chao, 1994; Hofstede et al., 2010). The reason we included regulation of learning and social media was that these two domains play an important role in university students’ life and are related to their well-being (Liu and Ma, 2018; Yang et al., 2019). On the one hand, it has been found that autonomous study not only positively predicted more adaptive learning and better academic performance, but also higher well-being among Chinese samples (Vansteenkiste et al., 2005). On the other hand, social media use, particularly excessive engagement, was negatively associated with university students’ subjective well-being (Ding et al., 2017). To enhance university students’ well-being and development, it is important to understand relations between autonomous supportive and psychologically controlling parenting styles and behavior regulation in these two major domains of their life. In addition, the potential mediating role of basic psychological needs has been argued to play a role in these relations (Vansteenkiste et al., 2020).

### Parental Autonomy Support, Parental Psychological Control, and Basic Psychological Needs

When living in a need-supportive environment, one’s needs are more likely to be satisfied (Ryan and Deci, 2017). Autonomy-supportive parents often provide children with informative feedback and meaningful choices instead of imposing control when they explore self-value and interests; therefore, children’s basic psychological need satisfaction is facilitated. For instance, children were found to be more persistent at problem-solving by themselves after interaction with autonomy-supportive mothers than with controlling mothers (Grolnick, 2002). Furthermore, Soenens and Vansteenkiste (2005) suggested that perceived autonomy enables children to master their world with an increased sense of competence (i.e., enhanced academic competence, social competence with peers and in job-searching behaviors). In addition, research has shown that autonomy support can strengthen children’s relatedness and closeness with others (Gurland and Grolnick, 2000; Fousiani et al., 2016). Importantly, the positive association between PAS and psychological need satisfaction has been supported within different cultural groups (Cordeiro et al., 2015; Inguglia et al., 2015; Zhou et al., 2019). Based on these findings, we therefore hypothesized that PAS would be positively related to basic psychological need satisfaction (BPNS) and negatively associated with basic psychological need frustration (BPNF) (Hypothesis 1).

Psychologically controlling parenting was associated with various negative consequences and its detrimental effects can be generalized across different cultures (Soenens and Vansteenkiste, 2010). Nevertheless, some researchers argued that psychologically controlling techniques are often used in Eastern cultures and their effects may be less maladaptive or even favorable (Chao, 1994; Wu et al., 2002). Cross-cultural studies have contradicted this claim. For example, Barber et al. (2005) investigated 10 nations and found universal adverse consequences of psychological control across these cultures. As for Chinese samples, similar results were obtained: parental psychological control was predictive of less adaptive learning strategies (Vansteenkiste et al., 2005) and depression and anxiety (Rudy et al., 2008).

In addition, parental psychological control was posited to associate with psychological need frustration since psychologically controlling strategies such as shaming and guilt induction are needs-thwarting in nature (Soenens and Vansteenkiste, 2010). That is, children often feel pressured to think and behave in order to seek approval and meet parents’ expectations. As a result, the autonomy need is directly dampened (Vansteenkiste et al., 2005). Furthermore, controlling parenting potentially deteriorates children’s capability such as time management, information processing and concentration, accordingly, impairing their self-belief in their competence (Soenens and Vansteenkiste, 2010). PPC also results in the deterioration of relatedness as long-term pressing makes children less connected to their parents and parents’ love may be perceived as conditional (Soenens and Vansteenkiste, 2010). Study of Costa et al. (2019) particularly pointed out the role of psychological control in the intergenerational transmission of need frustration: adolescents’ needs are often thwarted due to psychologically controlling parents who also experienced need frustration. Thus, we predicted that PPC would negatively impact BPNS and would be positively associated with BPNF (Hypothesis 2).

### Basic Psychological Need Satisfaction, Parental Autonomy Support and Autonomous Regulation of Learning

Learning is primarily an active process, and it is most favorable when pursued by intrinsic motivation, in which performed behavior is purely out of interest (Deci et al., 1996). However, learning can also be driven by extrinsic motivation, in which the purpose to perform an activity is for a separate outcome rather than inherent satisfaction of conducting the activity itself. In SDT, extrinsic motivation variables, including identified regulation (i.e., one identifies the importance of a behavior to the self) and integrated regulation (i.e., one fully integrates a motivation into the self-value) as well as intrinsic motivation are regarded as autonomous regulation because the behavior is volitional and valued by the self (Ryan and Deci, 2000). A wide variety of studies have demonstrated that autonomous regulation of learning (ARL) associates with positive outcomes such as more enjoyment of school, better academic achievement, and proactive coping with stress (e.g., Deci et al., 1996).

In SDT, BPNS is an important predictor of ARL. By maximizing students’ sense of volition and minimizing the sense of coercion, the experience of autonomy is enhanced, which facilitates intrinsic motivation or internalization (i.e., the process of taking the values of extrinsically motivated activities into personally endorsed regulation) and further predicts positive engagement and self-regulated learning behavior (Deci et al., 1996; Ryan and Deci, 2017). Studies of Zhen et al. (2017) and Wang and Tsai (2020) pointed out that one’s feeling of being competent to interact in the environment and conquer challenges can promote self-efficacy (i.e., one’s certain beliefs in the ability to successfully perform tasks or achieve goals) and academic engagement. Relatedness, especially connected to social partners such as teachers and parents, gives rise to the feeling of security and further flourishes one’s autonomous motivation to explore and learn (Zhen et al., 2017). Also, relatedness can promote autonomous regulation through internalizing others’ values and practices to whom learners feel connected (Deci et al., 1996). Accordingly, we predicted that BPNS would be positively related to ARL (Hypothesis 3).

In order to promote one’s self-regulated behavior, the importance of familial environments has been highlighted (Deci et al., 1996; Ryan and Deci, 2000). Autonomy-supportive parents promote ARL and academic performance of students at different stages. For example, the longitudinal study of Joussemet et al. (2008) emphasized the vital role of maternal autonomy support in 5-year-old pre-schoolers’ social and academic adjustment, while Grolnick and Ryan (1989), through in-depth structured interviews with parents, found that PAS was positively associated with school children’s self-reports of autonomous regulation.

The mediating role of need satisfaction in explaining the association between environment and students’ academic regulation, engagement and performance has been found in different sample groups. For example, perceived autonomy support facilitates Chinese primary school students’ need satisfaction, which further predicts their autonomous motivation and engagement (Zhou et al., 2019). Similar results were obtained among Grade 8 Australian physical education students (Garn et al., 2019) and university students in scientific subjects (Ratelle et al., 2005).

**FIGURE 1:**
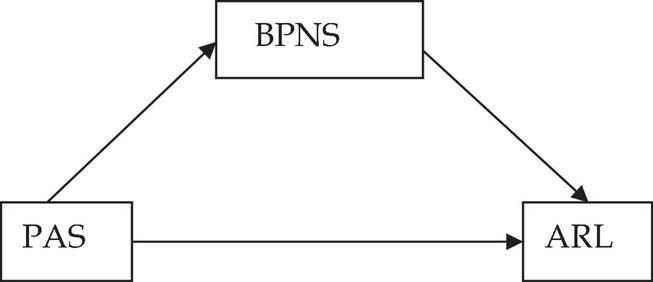
Hypothesized relationships between PAS, BPNS, and ARL. PAS, parental autonomy support; BPNS, basic psychological need satisfaction; ARL, autonomous regulation of learning.

However, studies examining the above-mentioned relations either heavily relied on samples of children and adolescents or were mainly conducted within individualist cultures, which center on the self. Researchers implied that these relations may fail to apply to people in collectivistic cultures as they attach importance to social obligations (Markus and Kitayama, 1991). One study supporting this claim found that autonomy was not significantly associated with learning engagement among Chinese students since they value more caring and harmonious relationships than autonomy and independence (Zhen et al., 2017). Moreover, little research has systematically examined these relationships within a sample of emerging adults (e.g., university students). According to Arnett (2000), emerging adulthood is a vital developmental stage from the late teens through the mid-to-late 20s and it is distinct in terms of demographic characteristics, subjective sense, and identity exploration (Arnett, 2004). Moreover, parental impact on young adults is different from that on adolescents (Inguglia et al., 2015). Therefore, how parenting practices associated with psychological needs and learning regulation of emerging adults in the collectivistic culture (i.e., China) was examined in this study. Built upon previous research, we estimated that PAS would increase students’ ARL through the mediating role of BPNS [their relation is illustrated in Figure 1 (Hypothesis 4)].

### Basic Psychological Need Frustration, Parental Psychological Control, and Dysregulation in Social Media Engagement

Apart from learning, university students nowadays devote a great portion of their time to social media. Despite the benefits of social media such as increased availability and accessibility of knowledge (Zhang and Xue, 2015) and the promotion of collective learning and interaction (Al-Rahmi and Othman, 2013), students’ excessive and compulsive engagement (i.e., obsessively and uncontrollably using social media at the expense of other activities) has drawn a wide concern (Andreassen, 2015). Incessant connection to social media has been found problematic and detrimental to well-being (Kross et al., 2013; Marino et al., 2017). Similar findings suggested this relation also applied to Chinese population, where students’ passive social media use undermined their well-being (Ding et al., 2017). Rooted in the SDT framework, research has suggested associations between psychological needs and compulsive online behavior (e.g., online gaming or social media engagement). For example, controlled internalization and *Fear of Missing Out* (i.e., the strong desire to have continuous connection with others not to miss out rewarding experience) were related to both deficits in psychological needs and dysregulated online behavior (Vallerand, 2008; Przybylski et al., 2013; Alt, 2015). That is, when basic needs are not satisfied, users are more likely to engage in online activities against their own volition. Thus, our fifth hypothesis was that BPNF would be positively related to DSME (Hypothesis 5).

More importantly, the mediating role of psychological needs has been identified between the relations of social environment and problematic online behavior among Chinese. For instance, stressful life events were related to Internet addiction, whose relation was mediated by psychological needs dissatisfaction among Chinese adolescents (Li et al., 2016). Autonomy support, on the other hand, was seen as a protective factor of Internet addiction (Liu et al., 2019) as it effectively enhances need satisfaction (Yu et al., 2015).

**FIGURE 2:**
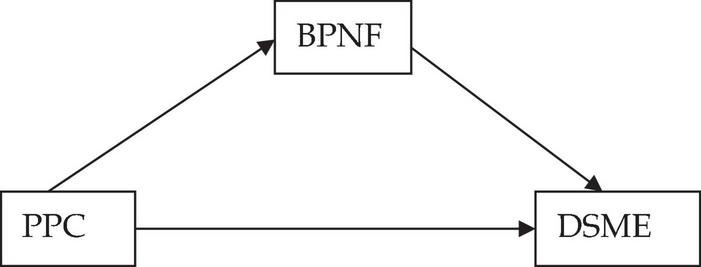
Hypothesized relationships between PPC, BPNF, and DSME. PPC, parental psychological control; BPNF, basic psychological need frustration; DSME, dysregulation in social media engagement.

Previous studies either focused on the relation between needs dissatisfaction and social media dysregulation (Przybylski et al., 2013; Alt, 2015) or the association between frustrated needs and addictive online gaming behavior (Mills et al., 2018; Tóth-Király et al., 2019; Chamarro et al., 2020). Plus, in terms of influence of controlling parenting on unhealthy Internet use, adolescents were the main focus of previous studies. Shek et al. (2018), for instance, pointed out both maternal and paternal psychological control could reinforce the excessive usage of the Internet, and fathers’ negative influences tended to last longer. However, research on relationships of parental psychological control, basic need frustration and dysregulation in social media engagement (DSME) among young adults is lacking. Therefore, the present study used the construct of DSME to describe the use of social media in an uncontrolled and non-self-determined manner at the cost of one’s well-being and aimed to fill this gap by investigating these associations among the three constructs. Our last hypothesis predicted that students with PPC are more likely to suffer from basic needs thwarting (BPNF), which in turn, led to DSME (see Figure 2); in other words, BPNF would mediate the relationship between PPC and DSME (Hypothesis 6).

### The Present Study

The Chinese culture is largely influenced by Confucian philosophy, which emphasizes respect for authority, devotion to parents, emotional restraint, and the importance of education (Russell et al., 2010). Some researchers argued that autonomy support may not have the same positive effects on children’s social functioning in Asian cultures as in the West (Chao and Tseng, 2002) and that psychologically controlling parenting may be less harmful because of the high emphasis on conformity and obedience to parents (Chao, 1994). However, SDT maintains that promotion of autonomy is beneficial to one’s well-being and behavior regulation whereas psychological control is detrimental (Ryan and Deci, 2017). Therefore, we aimed to investigate the specific roles of the two parenting styles in the domains of behavior regulation in learning and social media engagement and the potential mechanisms behind these relationships in a sample of Chinese university students.

## Materials and Methods

### Participants

To determine sample size, a two-tailed correlation power analysis was conducted. We set the value of α and *1 –*β to be 0.01 and 0.90 respectively, in order to achieve sufficient power and detect any effects (Field, 2013). *r* was set to be 0.25, which indicated a medium effect for correlation analysis (Cohen, 1992). A target sample size of 231 participants was obtained. To account for possible attrition, the sample size was increased by 30%. A total of 302 university students from southwest China took part in an online survey. Filtering out incomplete questionnaires resulted in 287 complete observations.

### Procedure

The survey was designed through *SurveyGizmo* and online for 2 weeks. Participants were sent the link of the survey and informed that the study aimed to investigate how parenting styles were related to their behavior regulation. They were notified that completing the questionnaire would take approximately 15 minutes and should feel no pressure to withdraw at any time. The voluntary, confidential, and anonymous nature of participation was assured. Then participants gave their consent before answering questions about their life and engagement in social media and were asked to choose their age group (i.e., under 18-year-old, 18 to 21, 22–24, and older than 24). To encourage participation, participants were given the opportunity to ask and have answered questions about the research.

All procedures performed in studies involving human participants were in accordance with the ethical standards of the Ethics Committee of University of Essex and with the 1964 Helsinki Declaration and its later amendments or comparable ethical standards.

### Materials

In addition to demographic information (age and gender), 64 questions from four different scales were selected (for an overview, see Supplementary Material). All items were translated into simplified Chinese by two speakers who were proficient in both Chinese and English. The researchers performed careful back translation to check and adjust the accuracy of original translation. All items were scored on a Likert-type scale from 1 (*strongly disagree*) to 5 (*strongly agree*). To justify the quality of these instruments, we measured coefficient θ in addition to coefficient α. Introduced by Armor (1974), coefficient θ can be highly applicable in situations where multidimensionality may exist and enjoys the benefits of easy understanding and computation (Teo and Fan, 2013).

#### Parental Autonomy Support and Parental Psychological Control

Parental autonomy support and parental psychological control were assessed by using subscales of the *General Parenting Style* (GPS) scale (Soenens et al., 2006). The questionnaire was slightly adapted to be equally applicable to participants of a single or two parent homes, or the parental representative(s) in their lives. PAS is composed of 7 items for each parent (e.g., “My mother/father listens to my opinion or perspective when I’ve got a problem”), whereas PPC of eight items for each parent (e.g., “My mother/father is always trying to change how I feel or think about things”). PAS score was computed using the average of maternal (*M* = 3.45, *SD* = 0.65) and paternal autonomy support (*M* = 3.36, *SD* = 0.67). Similarly, PPC scores were obtained from the average of psychological control of mother (*M* = 2.53, *SD* = 0.66) and father (*M* = 2.61, *SD* = 0.73). The internal consistencies of the GPS scale were good: α = 0.76 and α = 0.75 for maternal and paternal autonomy support; and α = 0.78 and α = 0.86 for psychological control of mother and father. Coefficient θ of PAS and PPC were θ = *0.86* and θ = 0.88, respectively.

#### Basic Psychological Need Satisfaction and Frustration

The basic psychological needs were tested using the 24-item basic psychological need satisfaction and frustration scale (BPNSFS) (Chen et al., 2015). This scale assessed six aspects with four items in each category: autonomy satisfaction (e.g., “I feel my choices express who I really am”; *M* = 3.09, *SD* = 0.81, α = 0.79); autonomy frustration (e.g., “I feel pressured to do too many things”; *M* = 2.36, *SD* = 0.74, α = 0.77); relatedness satisfaction (e.g., “I feel that the people I care about also care about me”; *M* = 3.51, *SD* = 0.84, α = 0.81); relatedness frustration (e.g., “I feel the relationships I have are just superficial”; *M* = 1.87, *SD* = 0.74, α = 0.79); competence satisfaction (e.g., “I feel capable at what I do”; *M* = 3.34, *SD* = 0.83, α = 0.84); and competence frustration (e.g., “I feel insecure about my abilities”; *M* = 2.22, *SD* = 0.80, α = 0.78). The overall BPNS and BPNF were calculated by averaging the three aspects on psychological need satisfaction and frustration, respectively. The coefficient θ also indicated high level of internal consistency of BPNS and BPNF: θ = 0.89 and θ = 0.88.

#### Dysregulation in Social Media Engagement

Dysregulation in social media engagement assessed how participants control and regulate themselves in terms of using social media. It was a self-designed 5-item measure loosely based on the obsessive passion subscale by Vallerand (2008) (e.g., “I feel like I must use social networking websites”). Items were averaged for all the social media users. The internal consistency for DSME was α = 0.76 and θ = 0.77.

#### Autonomous Regulation of Learning

The scale used was a slightly adapted version of the *Reasons for Learning Questionnaire* (RLQ) (Black and Deci, 2000). Only five items that represented autonomous regulation of learning of the original scale were used in this study (e.g., “I participate actively in my university courses, because I feel like it is a good way to improve my understanding of the material”). Item scores were averaged and the reliability (α = 0.62 and θ = 0.63) was considered acceptable (Ursachi et al., 2013; Daud et al., 2018).

### Data Analysis

Data were analyzed using *SPSS* statistical software (version 19.0). With regard to examination of correlates of parental practices, psychological needs and behavior regulation in learning and social media, researchers followed examples from previous studies (e.g., Przybylski et al., 2013). Firstly, bivariate correlations between observed variables were estimated; then we conducted two-step hierarchical regression models to control the effects of age and gender when evaluating primary hypotheses. Non-social media users were filtered out from the sample when analyzing dysregulated behavior. To test the direct and indirect mediation effects of BPNS and BPNF, a macro of SPSS called *PROCESS* was conducted, as it simplifies the mediation analyses with observed variables and is more powerful compared to commonly used Sobel’s test (Zhao et al., 2010; Hayes et al., 2017). Besides, *PROCESS* is based on bootstraping, a non-parametric resampling procedure, in which the assumption of normality of the sampling distribution is not imposed (Hayes et al., 2017).

## Results

### Preliminary Analyses

Preliminary analyses were conducted to determine gender and age effects. The age group by gender distribution is displayed in Table 1. Descriptive statistics and correlations among all the observed variables are shown in Table 2.

**TABLE 1:** Demographic characteristics of the study population.

	Gender	
	*Male*	*Female*
**Age Group**		
*Under 18-year-old*	2	1
*18–21*	109	33
*22–24*	60	58
*Older than 24*	14	10
**Total**	185	102

**TABLE 2:** Descriptive statistics and correlations of observed variables.

*Variables*	*M*	*SD*	*1*	*2*	*3*	*4*	*5*	*6*	*7*	*8*
*(1) Gender*			–							
*(2) Age*			0.21**	–						
*(3) PAS*	3.41	0.59	0.16**	0.28**	–					
*(4) PPC*	2.56	0.63	–0.09	−0.19**	−0.43**	–				
*(5) BPNS*	3.31	0.70	0.08	0.25**	0.51**	−0.20**	–			
*(6) BPNF*	3.85	0.65	−0.19**	−0.30**	−0.42**	0.37**	−0.37**	–		
*(7) DSME*	2.31	0.79	–0.00	–0.10	−0.15**	0.28**	–0.09	0.37**	–	
*(8) ARL*	2.62	0.62	0.00	0.08	0.18**	0.17**	0.19**	0.18**	0.27**	–

*Participants who did not complete General Parenting Style (GPS) Scale either due to a single parent or missing answers were removed. There were nine missing values for PAS- (n = 278) and eight missing values for PPC-subscales (n = 279). Participants who were non-social media users were filtered out.*

*PAS, parental autonomy support; PPC, parental psychological control; BPNS, basic psychological need satisfaction; BPNF, basic psychological need frustration; DSME, dysregulation in social media engagement; ARL, autonomous regulation of learning.*

Asterisks (**) mark the significance level p < 0.01.

There was a gender difference for both PAS (*r* = 0.16, *p* < 0.01) and BPNF (*r* = –0.19, *p* < 0.01). In other words, females perceived more parental autonomy support and experienced less psychological need frustration than males. Age was positively related to autonomy support (*r* = 0.28, *p* < 0.01) and need satisfaction (*r* = 0.25, *p* < 0.01), and negatively associated with psychological control (*r* = –0.19, *p* < 0.01) and need frustration (*r* = –0.30, *p* < 0.01). This suggests that as people grow older, the perceived parental autonomy support as well as their basic psychological needs fulfillment increases; on the other hand, participants may feel less psychologically controlled by their parents and less frustrated with their basic psychological needs with age.

### Primary Analyses

To test whether PAS is positively correlated to BPNS, and negatively related to BPNF (Hypothesis 1), BPNS and BPNF values were regressed onto PAS scores. Taking age and gender effects into account, results showed that PAS was a significant predictor of BPNS (β = 0.51, *p* < 0.001, *R*^2^ = 0.26, _95%_CI [0.40, 0.61]) and BPNF (β = –0.43, *p* < 0.001, *R*^2^ = 0.18, _95%_CI [–0.54, –0.32]). The results indicated that participants with autonomy supportive parents, irrespective of their gender and age, have higher levels of psychological need satisfaction and lower levels of psychological need frustration.

To test whether high levels of BPNS would be positively linked to ARL, scores of ARL were regressed onto the values of BPNS. Obtained results were in accordance with our third hypothesis, and participants who scored higher on BPNS had more ARL in their university courses than those with lower BPNS scores (β = 0.19, *p* < 0.01, *R*^2^ = 0.03, _95%_CI [0.07, 0.30]). In other words, participants whose basic psychological needs were satisfied tend to study in a more autonomous way.

**FIGURE 3:**
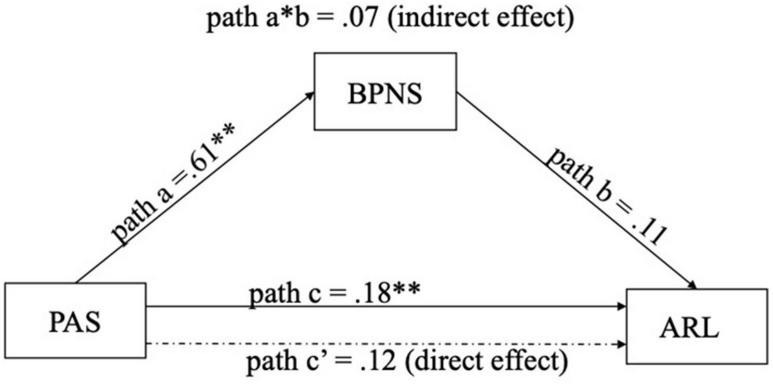
Relationships between BPNS, PAS, and ARL. BPNS, basic psychological need satisfaction; PAS, parental autonomy support; ARL, autonomous regulation of learning.

In Hypothesis 4, BPNS was predicted as a mediator of the relation between parental autonomy support and autonomous regulation of learning (see Figure 3).

The overall effect of PAS on ARL was significant, β = 0.18, *p* < 0.01, _95%_CI [0.07,0.30] (path c). Likewise, BPNS was predicted by PAS, β = 0.61, *p* < 0.001, *R^2^* = 0.26, _95%_CI [0.48,0.73] (path a). Considering the effect of PAS on BPNS in the relationship between BPNS and ARL, results did not show any significance, β = 0.11, *p* = 0.06, _95%_CI [–0.01,0.23] (path b). The indirect effect (a*b path) linking PAS and ARL *via* BPNS was not significant (β = 0.07, _95%_CI [–0.02,0.16]). The direct effect (path c’) on the association between PAS and ARL was not significant when taking BPNS into account (β = 0.12, *p* = 0.09, _95%_CI [–0.02,0.26]). Overall, results indicated that participants whose parents are autonomy-supportive tend to study in a more autonomous way, but this positive association was not through the mechanism of need satisfaction.

Parental psychological control was hypothesized to be negatively associated with BPNS but positively with BPNF. To examine the hypothesis, scores of BPNS were regressed onto PPC values (β = –0.20, *p* = 0.001, *R*^2^ = 0.04, _95%_CI [–0.32, –0.09]). When controlling for variability in the demographic data, the results still indicated a significant negative relationship (β = –0.16, *p* < 0.01, _95%_CI [–0.27, –0.04]). Age and gender accounted for 6.7% of the variance of psychological need frustration. The same analysis was performed to test the relationship between PPC and BPNF. Results showed a significant association between PPC and BPNF (β = 0.37, *p* < 0.001, *R*^2^ = 0.14, _95%_CI [0.26, 0.48]). Overall, these findings were in line with Hypothesis 2 and showed that the more psychologically controlling parents the participants had, the more likely they were to report lower levels of psychological need satisfaction and higher levels of need frustration.

Based on the assumption that BPNF positively correlated to DSME, scores of DSME were regressed onto BPNF values. Results indicated that when participants felt frustrated in their psychological needs, they were prone to have dysregulation in social media use (β = 0.35, *p* < 0.001, *R*^2^ = 0.12), which confirmed Hypothesis 5.

**FIGURE 4:**
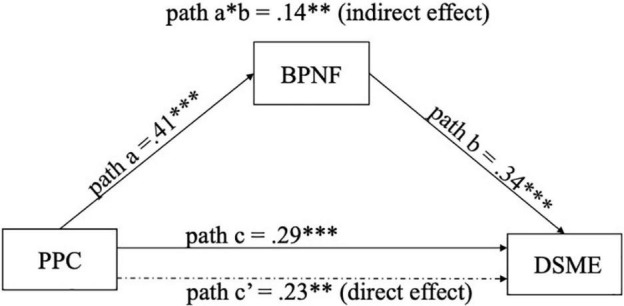
Relationships between BPNF, PPC, and DSME. PPC, parental psychological control; BPNF, basic psychological need frustration; DSME, dysregulation in social media engagement.

In Hypothesis 6, the link between PPC and DSME was predicted to be influenced by the mediating effect of BPNF (see Figure 4).

The association between PPC and DSME was significant (β = 0.29, *p* < 0.001, _95%_CI [0.22, 0.51]) (path c). Similarly, PPC was positively predictive of BPNF, β = 0.41, *p* < 0.001, _95%_CI [0.30, 0.53] (path a). Also, BPNF was associated with overuse of social media, β = 0.34, *p* < 0.001, _95%_CI [0.19, 0.49] (path b). The relationship between PPC and DSME, mediated by BPNF, showed a significant effect, β = 0.14, _95%_CI [0.07, 0.23] (a*b path). Furthermore, the direct effect between PPC and DSME (path c’) was significant when BPNF was considered, β = 0.23, *p* < 0.01, _95%_CI [0.07, 0.38]. Taken together, the results demonstrated that BPNF mediated the relationship between PPC and DSME. That is, psychologically controlling parenting can be either directly associated with students’ dysregulation in social media use or through the effect of frustrated psychological needs. The results from the mediation analysis are summarized in Table 3.

**TABLE 3:** Summary of mediation analysis (PROCESS).

	β	*p*	*BootLLCI*	*BootULCI*
***Mediation Analysis for BPNS, PAS and ARL***				
PAS predicting the mediator variable BPNS	0.61***	<0.001	0.48	0.73
PAS predicting the dependent variable ARL	0.18**	<0.01	0.07	0.30
BPNS predicting the dependent variable ARL	0.11	0.06	–0.01	0.23
Direct effect of PAS on ARL	0.12	0.09	–0.02	0.26
Indirect effect of PAS on ARL	0.07		–0.02	0.16
***Mediation Analysis for BPNF, PPC and DSME***				
PPC predicting the mediator variable BPNF	0.41***	<0.001	0.30	0.53
PPC predicting the dependent variable DSME	0.29***	<0.001	0.22	0.51
BPNF predicting the dependent variable DSME	0.34***	<0.001	0.19	0.49
Direct effect of PPC on DSME	0.23**	<0.01	0.07	0.38
Indirect effect of PPC on DSME	0.14**		0.07	0.23

*N = 278; Bootstrap sample size = 5000.*

*LLCI, low limit confidence interval; ULCI, upper limit confidence interval; PAS, parental autonomy support; PPC, parental psychological control; BPNS, basic psychological need satisfaction; BPNF, basic psychological need frustration; DSME, dysregulation in social media engagement; ARL, autonomous regulation of learning.*

Asterisks (**) mark the significance level p < 0.01 and (***) mark the significance level p < 0.001.

## Discussion

The present study addressed the roles of parental autonomy support and parental psychological control in Chinese emerging adults’ autonomous regulation in university studies and dysregulated engagement in social media. Our findings contribute to better understanding of young Chinese adults’ behavior regulation in several ways. First, perceived parental autonomy support and psychological control are related to students’ need satisfaction and frustration. Second, autonomy-supportive parenting facilitated autonomous learning. Third, psychologically controlling parenting was associated with the increase in uncontrolled use of social media. Finally, basic need frustration serves as an intervening variable between parental psychological control and dysregulation in social media use.

### Universal Influences of Parental Autonomy Support and Parental Psychological Control

Consistent with previous studies, our results confirmed the adaptive benefits of parental autonomy support and detrimental effects of parental psychological control on the three innate psychological needs in STD (Soenens and Vansteenkiste, 2010; Ryan and Deci, 2017). Perceived autonomy support helps university students to experience personal volition in their behavior, a sense of confidence and effectiveness in implementing given tasks and genuine closeness to one’s parents and other social figures (Hypothesis 1); in contrast, perceived parental psychological control impairs young adults’ basic needs for autonomy, competence, and relatedness (Hypothesis 2). Being forced to think and behave as dictated by controlling parents greatly thwarts one’s autonomy. Unable to solve problems in one’s own way, in the long term, one would experience insecurities and uncertainty about their competence; need for relatedness is also undermined because psychologically controlling parenting poses a threat to parent–child relationship, whose impairment further creates children’s social alienation.

In addition, our study demonstrated the universality of adaptive and maladaptive effects of these two parental dimensions (i.e., PAS and PPC) in the Chinese culture (Yu et al., 2018). Some research showing that autonomy support only benefits those who are in individualist culture is because autonomy support is conceived as promotion of independent act (Markus and Kitayama, 2003). However, PAS defined within STD emphasizes the promotion of volitional functioning rather than independence promotion (Joussemet et al., 2008; Soenens and Vansteenkiste, 2010). Autonomy supportive parents do not necessarily encourage their offspring to be independent (i.e., without parents’ support), but to act on their own volition (e.g., depend on or obedient to parents); as long as children behave based on self-endorsed motives, their needs will not be thwarted. Although Chinese students value interdependence, their basic needs are fulfilled if the reliance on others is practiced in a volitional way. In contrast, if parents promote dependence in a psychologically controlling way (e.g., inducing guilt to force children to stay in close proximity), which is against self-determination, the needs will be frustrated, regardless of the highly recognized cultural value of interdependence. In fact, when defined as the promotion of volition, cross-cultural studies have widely suggested the universal benefits of autonomy support and its relationship with psychological adjustment (e.g., academic motivation, Chirkov and Ryan, 2001; psychological well-being, Lan et al., 2019; intrinsic life goals, Lekes et al., 2010).

As for PPC, our study supported the generalization of its maladaptive characteristic by finding the positive association between PPC and need frustration. Compliance to parents and high academic achievement are considered of importance within Chinese culture and many Chinese parents use psychological controlling tactics to coerce children to remain in emotional and physical proximity or obtain high academic standards (Yu et al., 2018). However, the manipulative and pressuring feature of PPC can still frustrate universal needs for autonomy, competence, and relatedness (Soenens and Vansteenkiste, 2010; Soenens et al., 2012). Apart from the cross-cultural similarity, our study extended the literature by focusing on emerging adulthood, a period that is seen as extremely autonomous and influential in life (Arnett, 2004). Our findings confirmed that during this period, parenting styles still exerted significant impacts on young adults.

### Psychological Needs and Behavior Regulation in Learning and Social Media Use

The satisfaction of three basic psychological needs is essential to both intrinsic motivation and well-internalized regulation of behavior for learners (Deci et al., 1996; Ryan and Deci, 2017). Macakova and Wood (2020) found that satisfaction of the three needs, particularly autonomy was related to self-efficacy, which further associated with high academic achievement. Similar results were obtained among Taiwanese students, whose experience of need satisfaction was positively correlated to both general self-efficacy and science learning self-efficacy (Wang and Tsai, 2020). In fact, need satisfaction could significantly predict students’ learning engagement, which explained more than 70% of variance (Kurt and Tas, 2018).

An autonomy-supportive environment allows students to freely ask questions, openly share their opinions and express more interest in their studies (Reeve, 2012). Fazey and Fazey (2001) pointed out in their study that high levels of undergraduate students’ perceived competence were important to the promotion of autonomous learning. Furrer and Skinner (2003) found that when children’s sense of relatedness was fulfilled, they would experience less pressure and anxiety and engage in studies more actively.

Zhen et al. (2017) stated that autonomy satisfaction was not predictive of learning engagement; however, the reason for this conflicting result might be attributed to the characteristics of the research sample: Chinese middle school students. Chinese students in this stage still treat teachers as the authoritative organizers of courses and themselves as passive recipients of information; without relying on teachers, free exploration may elicit anxiety and shyness (Chen et al., 2008). Our study however, examined a sample of university students, who value autonomy need with increasing importance (Scabini, 2000) and perceive more autonomy support from others (Inguglia et al., 2015) compared to adolescents. Considering our findings that there was a positive association between BPNS and ARL (Hypothesis 3), one could speculate that when university students’ needs are fulfilled, they may be able to learn under their own volition.

Basic psychological needs were also found to be associated with the tendency to have excessive engagement in online activities. For instance, experience of need frustration was found to be related to addictive gaming (Mills et al., 2018; Tóth-Király et al., 2019; Chamarro et al., 2020). This is mainly because when people’s three innate needs are frustrated, they may display a tendency to seek rewards (i.e., extrinsic motives), act against their volition, and lose self-control, which were seen as compensatory behaviors to cope with need frustration (Vansteenkiste and Ryan, 2013; Carbonell and Panova, 2017). In line with previous results (Przybylski et al., 2013) that low levels of need satisfaction constituted a risk factor for dysregulated social media engagement, our findings emphasized the role of need frustration, the more harmful and destructive construct compared to low levels of need satisfaction, in excessive social media engagement, and corroborated its adverse effects on social media users (Hypothesis 5).

### Mediating Role of Basic Psychological Needs

The mediating role of need satisfaction in the association between autonomy-supportive environment and desirable outcomes in education has been widely researched (Ratelle et al., 2005; Joussemet et al., 2008; Zhou et al., 2019). However, our findings showed no significant mediating effect of need satisfaction for parental autonomy support and self-determined regulation in learning (Hypothesis 4). A possible explanation for the absence of an effect may relate to omitted mediators. According to Zhao et al. (2010), if a model has an insignificant a*b path (i.e., the indirect effect linking PAS and ARL *via* BPNS was not significant), but a significant c path (i.e., the overall effect of PAS on ARL was significant), the insignificance often results from undiscovered mediators. Based on previous research (Lekes et al., 2010; Wang and Tsai, 2020), we suggest that intrinsic life goals and self-efficacy as multiple mediators for further investigation. When parents are perceived as autonomy encouraging, students are more likely to dedicate themselves to intrinsic life goals and hold strong beliefs in their own ability to complete learning tasks, which may further link to self-determined regulation of learning.

Based on the positive relation between DSME and BPNF, we further considered PPC, as the antecedent to the maladaptive outcome, since need frustration often arises from controlling parenting (Costa et al., 2019). Soenens and Vansteenkiste (2010) also suggested that basic psychological needs can be the mechanism explaining the association between psychological control and maladjustment among children and adolescents. Following this speculation, previous studies emphasized the mediating role of the lack of need satisfaction between social context and uncontrolled online behaviors such as Internet addiction (Li et al., 2016) and problematic online game use (Yu et al., 2015). One study from Mills et al. (2018) focused on the severer status of need frustration and found that obsessive passion for gaming and time spent on gaming were related through impaired basic needs. Our results were in line with these studies and further extended their findings by investigating the specific mediating relation of parental psychological control, dysregulated social media use and need frustration among a sample of Chinese university students. That is, students with psychologically controlling parents whose basic needs are impaired have a greater tendency to have compulsive tendency in using social media, which supports Hypothesis 6. However, our partially mediating model also implies that other mechanisms (e.g., lack of control and self-criticism) may exist to explain the link between controlling parents and maladaptive outcomes (Scharf and Goldner, 2018).

## Limitations and Future Research Directions

Several limitations and future directions for further research must be noted. First, additional variables related to need satisfaction such as life goals, self-efficacy and passion should be included to expand the model tested (Vallerand, 2008; Lekes et al., 2010; Zhen et al., 2017; Wang and Tsai, 2020).

Second, despite certain directions between variables having been postulated, the correlational nature of our study fails to draw causal inferences and many of the relations might be reciprocal. For example, Soenens et al. (2008) found maladjustment of adolescents to be the predictor of psychologically controlling parenting. Therefore, future studies could examine whether students’ need satisfaction and autonomous regulation would cause their parents to give them more autonomy, and whether their frustrated needs and compulsive behavior could make parents use more psychologically controlling strategies.

Third, multiple methodological approaches such as experimental and qualitative methods are suggested to verify the results in various ways, as we only used self-report to measure the research constructs.

Four, regarding the lower reliability of ARL, compared to other scales, we speculated that this may be due to misunderstanding of some questions in the ARL subscale arisen from translation and cultural differences. For example, the word “challenge (挑战)” (item 5 of *Autonomous Regulation of Learning* scale, see Supplementary Material) could have been interpreted as referring to obstacles and difficulties, rather than self-enhancement.

Despite its limitations, the present study has both theoretical and practical implications. Theoretically, our study reemphasized the universal benefits of parental autonomy support to emerging adulthood and provided evidence on the detrimental effects of psychologically controlling parents on students’ malfunctioning regulation in the domain of social media involvement.

Regarding practical implications, our results suggest that parents should be made aware by schools and universities of the vital importance of being autonomy supportive to their offspring, even during the period when they are becoming adults. They should take students’ perspectives and acknowledge their feelings, especially when they are in stressful transition period; they should also encourage them to make decisions on their own without imposing ideas. Also, in order to encourage students to use social media in a healthy way, parents should realize that creating a psychologically controlling environment is counterproductive. When designing interventions to encourage autonomous learning and healthy social media use, researchers might want to take into account parenting styles and students’ psychological needs.

Taken together, our findings add to the body of knowledge on the relationship between parenting styles and basic psychological needs, as well as on the associations they have with students’ regulation in learning and social media use. They clearly show that autonomy-supportive parenting could benefit needs fulfillment and promote autonomous learning, whereas psychologically controlling parenting would thwart the basic needs, thereby linking to excessive social media usage.

## Data Availability Statement

The raw data supporting the conclusions of this article are available from the corresponding author on reasonable request.

## Ethics Statement

All procedures performed in this study were in accordance with the ethical standards of the University of Essex Research Committee and with the 1964 Helsinki Declaration and its later amendments. The studies involving human participants were reviewed and approved by the University of Essex Research Committee. Written informed consent to participate in this study was provided by the participants online.

## Author Contributions

SW was responsible for conception, data collection and analysis, interpretation and drafting of the manuscript. AL contributed to the study design, interpretation and drafting of the manuscript. AL, TT, and AM contributed to revising the article critically. All authors approved the submitted version of the article.

## Conflict of Interest

The authors declare that the research was conducted in the absence of any commercial or financial relationships that could be construed as a potential conflict of interest.

## Publisher’s Note

All claims expressed in this article are solely those of the authors and do not necessarily represent those of their affiliated organizations, or those of the publisher, the editors and the reviewers. Any product that may be evaluated in this article, or claim that may be made by its manufacturer, is not guaranteed or endorsed by the publisher.
