# Supplementary material for: Parental Autonomy Support, Parental Psychological Control and Chinese University Students’ Behavior Regulation: The Mediating Role of Basic Psychological Needs
Source: Front Psychol. 2022 Feb 18;12:735570. doi: 10.3389/fpsyg.2021.735570 (PMC8895294; doi:10.3389/fpsyg.2021.735570)
Supplement: Supplementary file 1 [file Data_Sheet_1.pdf]

Parental Autonomy Support, Parental Psychological Control and Chinese University Students' Behavior Regulation: The Mediating Role of Basic Psychological Needs

Songqin Wei, Timothy Teo, Anabela Malpique, Adi Lausen

Instructions

Below is a collection of statements about your everyday experience. Using the scale provided please indicate how true each statement is for you given your general experiences. Please answer according to what really reflects your experience rather than what you think your experience should be. Please treat each item separately from every other item.

Anchors

Strongly disagree	|	1
Disagree	|	2
Neither agree nor disagree	|	3
Agree	|	4
Strongly agree	|	5

Basic Psychological Need Satisfaction and Frustration

Autonomy Satisfaction

1. I feel a sense of choice and freedom in the things I undertake
2. I feel that my decisions reflect what I really want
3. I feel my choices express who I really am
4. I feel I have been doing what really interests me

Autonomy Frustration

5. Most of the things I do feel like "I have to"
6. I feel forced to do many things I wouldn't choose to do
7. I feel pressured to do too many things
8. My daily activities feel like a chain of obligations

Relatedness Satisfaction

9. I feel that the people I care about also care about me
10. I feel connected with people who care for me, and for whom I care
11. I feel close and connected with other people who are important to me
12. I experience a warm feeling with the people I spend time with

Relatedness Frustration

13. I feel excluded from the group I want to belong to
14. I feel that people who are important to me are cold and distant towards me

15. I have the impression that people I spend time with dislike me
16. I feel the relationships I have are just superficial

Competence Satisfaction

17. I feel confident that I can do things well
18. I feel capable at what I do
19. I feel competent to achieve my goals
20. I feel I can successfully complete difficult tasks

Competence Frustration

21. I have serious doubts about whether I can do things well
22. I feel disappointed with many of my performance
23. I feel insecure about my abilities
24. I feel like a failure because of the mistakes I make

Dysregulaiton in Social Media Engagement

1. I have a tough time controlling my need to use the Internet
2. I struggle to stop using the Internet
3. I feel like I must use social networking websites
4. I find myself using social networking websites automatically, without being aware I'm doing it
5. When I use social networking sites I sometimes feel like I am "running on automatic"

Autonomous Regulation of Learning

A. I participate actively in my university courses:

1. Because I feel like it is a good way to improve my understanding of the material
2. Because a solid understanding of coursework is important to my intellectual growth

B. I am likely to follow my instructor's suggestions for studying

3. Because he/she seems to have insight about how best to learn course material

C. The reason that I will work to expand my knowledge in my university courses:

4. Because it is interesting to learn more about the nature (underlying aspects/laws) of my course of study
5. Because it is a challenge to really understand how to solve problems

Parental Autonomy Support and Parental Psychological Control

Parental Autonomy Support mother

1. My mother listens to my opinion or perspective when I've got a problem
2. My mother is usually willing to consider things from my point of view
3. Whenever possible, my mother allows me to choose what to do
4. My mother allows me to decide things for myself
5. My mother insists upon doing things her way
6. My mother isn't sensitive to many of my needs
7. My mother helps me to choose my own direction in life

Parental Psychological Control mother

8. My mother is always trying to change how I feel or think about things
9. My mother changes the subject whenever I have something to say
10. My mother often interrupts me
11. My mother blames me for other family members' problems
12. My mother brings up past mistakes when she criticizes me
13. My mother is less friendly with me if I do not see things her way
14. My mother will avoid looking at me when I have disappointed her
15. If I have hurt her feelings, my mother stops talking to me until I please her again

Parental Autonomy Support father

16. My father listens to my opinion or perspective when I've got a problem
17. My father is usually willing to consider things from my point of view
18. Whenever possible, my father allows me to choose what to do
19. My father allows me to decide things for myself
20. My father insists upon doing things his way
21. My father isn't sensitive to many of my needs
22. My father helps me to choose my own direction in life

Parental Psychological Control father

23. My father is always trying to change how I feel or think about things
24. My father changes the subject whenever I have something to say
25. My father often interrupts me
26. My father blames me for other family members' problems
27. My father brings up past mistakes when he criticizes me

28. My father is less friendly with me if I do not see things his way
29. My father will avoid looking at me when I have disappointed him
30. If I have hurt his feelings, my father stops talking to me until I please him again
